# Fuzzy set-based generalized multifactor dimensionality reduction analysis of gene-gene interactions

**DOI:** 10.1186/s12920-018-0343-0

**Published:** 2018-04-20

**Authors:** Hye-Young Jung, Sangseob Leem, Taesung Park

**Affiliations:** 1 Faculty of Liberal Education, Seoul National University, Seoul, 08826 South Korea; 2 Department of Statistics, Seoul National University, Seoul, 08826 South Korea

**Keywords:** Gene-gene interaction, Fuzzy-set theory, FGMDR, Multifactor dimensionality reduction

## Abstract

**Background:**

Gene-gene interactions (GGIs) are a known cause of missing heritability. Multifactor dimensionality reduction (MDR) is one of most commonly used methods for GGI detection. The generalized multifactor dimensionality reduction (GMDR) method is an extension of MDR method that is applicable to various types of traits, and allows covariate adjustments. Our previous Fuzzy MDR (FMDR) is another extension for overcoming simple binary classification. FMDR uses continuous member-ship values instead of binary membership values 0 and 1, improving power for detecting causal SNPs and more intuitive interpretations in real data analysis. Here, we propose the fuzzy generalized multifactor dimensionality reduction (FGMDR) method, as a combined analysis of fuzzy set-based analysis and GMDR method, to detect GGIs associated with diseases using fuzzy set theory.

**Results:**

Through simulation studies for different types of traits, the proposed FGMDR showed a higher detection ratio of causal SNPs, compared to GMDR. We then applied FGMDR to two real data: Crohn’s disease (CD) data from the Wellcome Trust Case Control Consortium (WTCCC) with a binary phenotype and the Homeostasis Model Assessment of Insulin Resistance (HOMA-IR) data from Korean population with a continuous phenotype. The interactions derived by our method include the pre-reported interactions associated with phenotypes.

**Conclusions:**

The proposed FGMDR performs well for GGI detection with covariate adjustments. The program written in R for FGMDR is available at http://statgen.snu.ac.kr/software/FGMDR.

## Background

In many genetic association studies, despite successful identification of genetic factors that govern various phenotypes, parts of heritability remained unexplained as the ‘missing heritability’ [1]. For example, heritability of height is assessed 55–81% [2, 3] and about 40 SNPs are discovered by their association with the height. However, these genetic variations explain only 5% of height variation [4]. To explain missing heritability, many studies have been proposed and performed, including large sample-size studies to detect weak effect SNPs [5], next-generation sequencing techniques have been used to overcome design flaws of SNP chips, such as rare variant detections [6]. Epigenetic factors and population stratification can be other sources of missing heritability [7].

Among efforts to explain the missing heritability, the analysis of gene-gene interactions (GGIs) has been studied to understand the etiology of common complex traits using statistics and machine learning [8]. Among the many different machine learning approaches for detecting GGIs, multifactor dimensionality reduction (MDR), proposed by Ritchie et al. [9] has received much interest, and numerous extensions of MDR have been now developed, including quantitative MDR, for quantitative traits [10]; generalized MDR (GMDR), for both quantitative and binary traits [11]; MB-MDR, based on statistical testing [12], Surv/Cox-MDR, for survival data [13, 14]; FAM-MDR, for family data [15], GEE/Multi-MDR, for multivariate traits [16, 17], etc.

Among the many extensions of MDR, GMDR tests GGIs using residuals of a generalized linear model as score statistics. This idea permits adjustment of covariates, addressing both binary and continuous phenotypes [11]. In many genome-wide association studies (GWASs), data consists of thousands or more samples, and information on each sample consists not only of genetic information, but also non-genetic information, such as age, sex, and weight. In these cases, the significances of SNPs can be different whether or not non-genetic information is used as covariates. In other words, some phenotype associated SNPs can be hidden, and some non-causal SNPs can be discovered in analysis, without covariate adjustments. Additionally, recent genetic association studies [18] consist of multiple ethnic groups. In these cases, analysis without concerning about population stratification, produces misleading results [19] and principle components can be used as covariates for adjusting of the population stratification [20]. Therefore, covariate adjustment is essential for analysis, in genetic association studies.

Including GMDR, MDR-based methods reduce a dimensionality of genetic information of multiple SNPs to one dimension with binary values (high-risk: H or low-risk: L). This basic idea of MDR, makes all binary interaction models detectable and potentially extending to numerous types of data and methods. However, these extensions, like MDR frameworks, are based on traditional classifications that allow each genotype combination to belong to only one of high/low risk groups. In traditional classification, the class membership value is binary, and an object is a member of a class or not. Such traditional classification may not reflect real phenomena in biological and medical studies, because traditional classification approach can imply the following shortcomings. On one hand, genetic variants with similar characteristics can be classified into different risk groups. On the other hand, genetic variants with different characteristics can be classified into the same risk groups. For example, in GMDR [11], each cell is assigned as H or L based on whether its score is higher or lower than a threshold. Thus, some cells near the threshold are classified into different groups, despite similar scores. Additionally, cells in the same group (H or L) are considered the same, despite having different scores. Unlike GMDR, QMDR [10] tests the significance of a cell using quantitative trait differences between cases and controls. This concept resembles MB- MDR [12] using ternary classification. However, although binary classification extends to ternary classification, its sufficiency is still a question. In other words, the shortcomings of traditional classification methods, mentioned above, still remain.

The fuzzy set, introduced by Zadeh [21], handles these shortcomings, caused by traditional classification, through allowing partial membership of H and L groups. In the example mentioned above, membership values of a cell near a threshold can be 0.6 for H group and 0.4 for L group. Likewise, membership values of two cells, having different scores in the H group can be different as follows: one can be 0.2 for H group (0.8 for L group) and the other can be 0.8 for H (0.2 for L group), respectively. Fuzzy clustering and fuzzy neural network as machine learning approaches, are well known, with many successful applications in medicine [22], finance [23], image processing and engineering [24]. In bioinformatics, some studies based on fuzzy set theory, have been introduced, but not actively studied, until now [25, 26].

In our previous study [27], we were the first to propose Fuzzy MDR (FMDR) framework to detect GGIs in the context of binary trait, and demonstrated that FMDR has a higher power than the original MDR. FMDR based on fuzzy classification, allows the partial membership of high and low risk groups, and as such can overcome drawbacks due to traditional classification which are not well explained thoroughly by using original MDR. Through real application to bipolar disorder (BD) data of Wellcome Trust Case Control Consortium (WTCCC) [28], we identified two-loci SNP combinations associated with BD [27]. Since Fuzzy MDR analysis based on fuzzy classification provides different levels of membership degrees of H/L for each cell, more flexible interpretations for results are possible. To that end, we showed that simple pattern analysis allowed us to match FMDR results to well-known biological epistasis models [27]. However, FMDR, like MDR, can only deal with binary traits, and does not allow covariate adjustment.

In this paper, we propose fuzzy set-based generalized multifactor dimensionality reduction (FGMDR) to detect GGIs while allowing for covariate adjustment. Since FGMDR is based on the generalized linear models, it can be applied to both quantitative and binary traits. FGMDR serves as a generalized MDR framework, including Fuzzy MDR, MDR, and GMDR. Through simulation studies with different epitasis models, as listed by Velez et al. [29], we compare the power of FGMDR to that of GMDR and MDR.

The remainder of this paper is organized as follows: the GMDR framework is briefly reviewed, and the algorithm of FGMDR is proposed. The power of the proposed FGMDR, using several simulations under different epitasis models, is presented. We then present the results of FGMDR applied to Crohn’s disease (CD) dataset and a homeostatic model assessment of insulin resistance (HOMA-IR) dataset. Finally, the results are discussed and put into logical context.

## Methods

### Review of GMDR

Lou et al. [11] proposed a GMDR framework, based on the score of a generalized linear model, as follows. Let *y*_*i*_denote the phenotype of individual i with expectation *E*(*y*_*i*_) = *μ*_*i*_. In general, this can be represented by the following generalized linear model (GLM): $l\left({\mu}_i\right)=\alpha +{x}_i^T\beta +{z}_i^T\gamma$ where *l*(*μ*_*i*_) is the link function, *α* is the intercept, and *x*_*i*_ is a vector that expresses possible genotype combinations of interest. The variable *z*_*i*_ is a vector representing environmental factors, and *β* and *γ* are coefficient vectors. In the first step, the residual based on GLM, is calculated from the null model *β* = 0. At the second step, the average value of residuals is calculated within each multifactor cell of contingency table to classify each SNP combination. Cells are then classified either as “high risk group H”, if the average value is nonnegative (or meets or exceeds a preassigned threshold T) or as “low risk group L”, if the average value is negative (or does not exceed threshold T). At the third step, the balanced accuracy (*BA*) is calculated using the sum of residuals. Through a 10-fold cross-validation, the best *k*-way model having the minimum prediction error and maximum cross-validation consistency is selected.

GMDR framework is based on traditional classification, allowing each genotype combination to belong to only one of H/L groups. However, this classification may not reflect characteristics of genotype combinations corresponding to “tied” cells. To overcome this drawback, fuzzy set allows for partial membership for H/L groups, in the GMDR framework.

### The proposed FGMDR

The fuzzy set proposed by Zadeh has been employed to handle the concept of partial membership of elements in a set [21]. The only difference between a classical set and a fuzzy set is the range of the membership values. A classical set has its membership value in the set [22] while a fuzzy set has its membership value in the interval [0,1]. Since each genotype combination cannot be divided sharply into H/L groups, a fuzzy set which sees the world in shades of gray may be more appropriate to represent the real biological phenomena. A fuzzy set A in the universal space X is a set of ordered pairs {(*x*, *μ*_*A*_(*x*)) | *x* ∈ *X* }, where *μ*_*A*_(*x*) on [0,1] represents the “degree of membership” of *x* in the fuzzy set *A*. When *A* is a classical set, its membership value is to be 1 or 0, as to whether or not an element is a member of a set. Thus, a classical set is considered a special case of a fuzzy set. GMDR, therefore, uses the traditional classification based on classical set, to reduce the dimensionality of genotype combinations, by grouping cells into H/L groups. By adopting the fuzzy set theory, we propose an FGMDR representing H/L groups by two fuzzy sets, which are identified by the membership functions *μ*_*H*_ and *μ*_*L*_, respectively. By introducing these membership functions, FGMDR allows each genotype combination to partially belong to both H/L groups, while GMDR restricts each genotype combination to belong to only one of H and L groups.

For the phenotype *y*_*i*_ for individual *i*, GMDR uses a studentized (standardized by a sample-based estimate of a population standard deviation) residual based on GLM as a score for GMDR, as follows: *S*_*i*_*=* $\frac{\left({y}_i-{\widehat{\mu}}_i\right)}{\sqrt{\widehat{Var}\left({y}_i-{\widehat{\mu}}_i\right)}}$ where ${\widehat{\mu}}_i$ is the estimated expectation and $\widehat{Var}\left({\mathrm{y}}_{\mathrm{i}}-{\widehat{\mu}}_i\right)$ is the estimated variance of residual.

For a dataset having *p* SNPs, let ${S}_{\bullet}^j$ be the average value of scores within the j-th multifactor cell, where *j* = {1, ⋯, 3^*k*^},  *k* is a number of SNPs in an interaction model (interaction order). Since GMDR uses balanced accuracy based on classical sets of H/L groups having membership values 0 and 1, the magnitude of the value ${S}_{\bullet}^j$ within the j-th multifactor, is ignored. This is a motivation of the proposed FGMDR. In FGMDR, we consider a sigmoid membership function with respect to ${S}_{\bullet}^j$ given by

1 $$ {\mu}_H\left({S}_{\bullet}^j\right)=\left\{\begin{array}{cc}0& {S}_{\bullet}^j<{t}_l\\ {}\frac{1}{1+{\left(\frac{S_{\bullet}^j-{t}_h}{S_{\bullet}^j-{t}_l}\right)}^2}& {t}_l\le {S}_{\bullet}^j<{t}_h\\ {}1& {S}_{\bullet}^j\ge {t}_h\end{array}\right.,{\mu}_L\left({S}_{\bullet}^j\right)=1-{\mu}_H\left({S}_{\bullet}^j\right) $$

In the above membership functions (1), the two threshold values *t*_*l*_, *t*_*h*_ (*t*_*l*_ ≤ *t*_*h*_) need to be determined a priori.

From fuzzy set theory, the following measures can be computed:

$$ {TP}_{FUZZY}=\sum \limits_j{S}_{+1}^j{\mu}_H\left({S}_{\bullet}^j\right),{FN}_{FUZZY}=\sum \limits_j{S}_{+1}^j{\mu}_L\left({S}_{\bullet}^j\right),{FP}_{FUZZY}=\sum \limits_j{S}_{+0}^j{\mu}_H\left({S}_{\bullet}^j\right),{TN}_{FUZZY}=\sum \limits_j{S}_{+0}^j{\mu}_L\left({S}_{\bullet}^j\right) $$

where ${S}_{+0}^j$ is the sum of negative score values within the *j*-th multifactor cell, and ${S}_{+1}^j$ is the sum of nonnegative score values within the *j*-th multifactor cell. Then, the balanced accuracy (*BA*) using the membership function, *BA*_*FUZZY*_, is defined as the arithmetic mean of *SEN*_*FUZZY*_ and *SPE*_*FUZZY*_ introduced in [27]. In the proposed FGMDR, we use *BA*_*FUZZY*_ as an evaluation measure to detect the best interaction model. Note that *BA*_*FUZZY*_ reduces to the *BA* value used in the GMDR, when an indicator function is used as the membership function. Thus, the FGMDR method shares the same framework as FMDR [27], except for replacing the case-control ratios by the sum of residuals in each cell.

## Results

First, we compared MDR, FMDR, GMDR, and FGMDR in terms of their success rates (power) for causal SNPs detection, using various simulation data consisting of a continuous phenotype category and two case-control categories. In simulation data experiments, the FGMDR showed higher power than the others; consequently, we applied FGMDR to two real datasets for illustrations.

### Simulation study

Simulation data consists of three categories of data, one continuous phenotype and two case-control categories. In the continuous phenotype categories (scenario 1), a phenotype variable is calculated by a linear sum of genetic effects, covariates, and error terms, to simulate a continuous phenotype such as blood pressure. In the first case-control category (scenario 2), a binary variable is 1 or 0, depending on whether or not a continuous value exceeds a certain threshold value. This type of data is generated for simulation of diseases whose status is determined by continuous variables, such as obesity. In the second case-control category (scenario 3), a binary variable is determined as a probability, based on a logit model with genetic effects, covariates, and error terms, for simulation of binary type diseases such as cancer.

Common to all three scenarios, genetic effects are based on 70 penetrance models (7 heritability values: 0.01~ 0.4, 2 minor allele frequency values: 0.2, 0.4 and 5 interaction models), without marginal effect [29], and power is defined as a proportion of how many times the true causal SNPs were selected as the model with the highest BAs (BA for MDR and GMDR, *BA*_*FUZZY*_ for FMDR and FGMDR) among 100 replicates for a given model. Each replicate consists of 2000 samples (1000 cases and 1000 controls for case-control types), with genotype information for 100 SNPs, a covariate, and a phenotype. Genotype values of two causal SNPs are then determined by the minor allele frequency (MAF: 0.2, 0.4) of the penetrance models, and genotype values of non-causal SNPs, are randomly selected from a Hardy-Weinberg equilibrium, based on MAF values in [0.05, 0.5]. We tested various coefficients of disease models. In the results from tests, consistent patterns were seen. Therefore, in each scenario, coefficients of disease models are adjusted to about a 50~ 60% average success rate for all the methods.

### Scenario 1

**Fig. 1:**
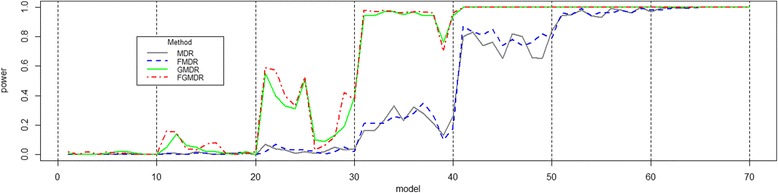
Power comparison of scenario 1 data for a continuous phenotype. The powers of MDR and FMDR are lower than that of others, because they cannot use information in covariates. FGMDR shows higher power than that of GMDR, in some penetrance models, or similar power. In terms of the average power, MDR was 0.427, FMDR was 0.433, GMDR was 0.611 and FGMDR was 0.621

Since scenario 1 simulates a continuous phenotype, we used the following model with an identity link function *Y*_*i*_ = α + *X*_*i*_^*T*^*β* + *Z*_*i*_^*T*^*γ* + *ε*_*i*_, where *Y*_*i*_ represents a phenotype value, *X*_*i*_ represents a genetic effect, *Z*_*i*_ represents covariates of the *i*th individual, and *ε*_*i*_ represents the error. *X*_*i*_ is randomly selected based on normal distribution of the mean: a penetrance value corresponding to the genotype value of the *i*th individual and standard deviation 0.1. *Z*_*i*_ is randomly selected on a normal distribution of mean 0, and standard deviation 0.7. *ε*_*i*_ is randomly selected on a normal distribution of mean 0, and standard deviation 1. All values of coefficients (*β*, *γ*) are the same as one. Simulation results of scenario 1 are summarized in Fig. 1.

In Fig. 1, the powers of MDR and FMDR are lower than that of others, because they cannot use information in covariates. In other words, covariates are useful for causal SNP detections in genetic association studies. Then, FGMDR shows higher power than that of GMDR, in some penetrance models, or similar power. In terms of the average power, MDR was 0.427, FMDR was 0.433, GMDR was 0.611 and FGMDR was 0.621. Additionally, we performed significance testing of power comparisons, using the Wilcoxon signed-rank test. The power of FGMDR is significantly higher than that of MDR (*p*-value: 2.82e-10), FMDR (p-value: 5.99e-11 and GMDR (p-value: 2.59e-02).

### Scenario 2

**Fig. 2:**
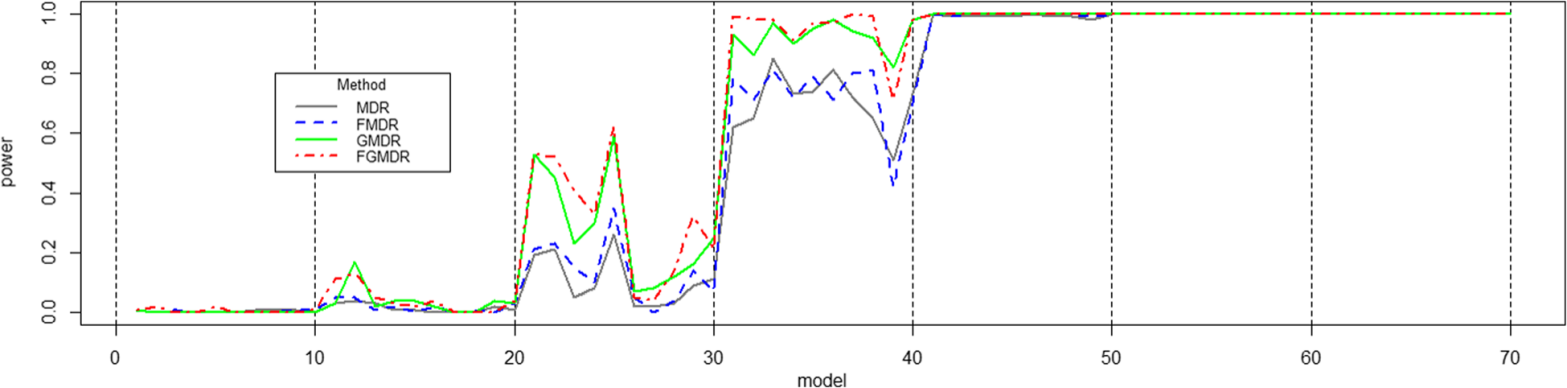
Power comparison of scenario 2 data for a binary phenotype derived from a continuous value. The powers of the MDR and FMDR, for scenario 2, are lower than that of others in many penetrance models, and it means importance of covariates in case-control association studies. Among the other two methods, the FGMDR showed higher than that of GMDR, in some penetrance models. In terms of the average power, MDR was 0.545, FMDR was 0.555, GMDR was 0.606 and FGMDR was 0.616

Since scenario 2 simulates a binary phenotype determined by a continuous value, we calculated a continuous value, and discretized as 1 for higher than a specific threshold (a median of the continuous values), or 0 for the others. For a continuous value calculation, we used the same identity link function in GLM as scenario 1. All other parameter values, except the standard deviation of error (0.5), are the same as in scenario 1. The simulation results of scenario 2 are summarized in Fig. 2.

In Fig. 2, similar patterns as in Fig. 1, are shown. The powers of the MDR and FMDR, for scenario 2, are lower than that of others in many penetrance models, and it means importance of covariates in case-control association studies. Among the other two methods, the FGMDR showed higher than that of GMDR, in some penetrance models. In terms of the average power, MDR was 0.545, FMDR was 0.555, GMDR was 0.606 and FGMDR was 0.616. Wilcoxon signed-rank tests showed that the mean power of FGMDR was significantly higher than that of MDR (*p*-value: 1.35e-07), FMDR (p-value: 2.26e-06) and also higher than that of GMDR, but not significantly (p-value: 5.15e-02).

### Scenario 3

Since scenario 3 simulates a binary phenotype with a probability using a logit model given below:

$$ \ln \left(\frac{p\left({Y}_i=1\right)}{1-p\left({Y}_i=1\right)}\right)=\upalpha +{X_i}^T\beta +{Z_i}^T\gamma +{\varepsilon}_i. $$

**Fig. 3:**
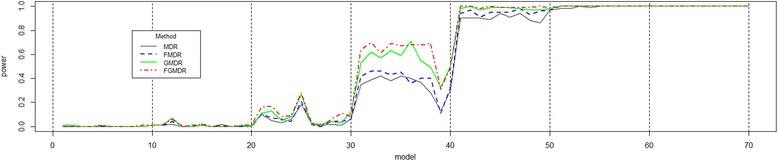
Power comparison of scenario 3 data for a binary phenotype generated from a logit model. The order of power (MDR < FMDR < GMDR < FGMDR) was consistently similar with previous results, and the power of all the methods increased in both the heritability and MAF values. In terms of the average power, MDR was 0.473, FMDR was 0.487, GMDR was 0.519, and FGMDR was 0.533

In this scenario, the value of *β* is reduced to 0.5, and the standard deviation of the error increased to 2. The simulation results of scenario 3 are summarized in Fig. 3.

The simulation results of scenario 3 in Fig. 3 show some interesting patterns, compared to the previous results. Here, the order of power (MDR < FMDR < GMDR < FGMDR) was consistently similar with previous results, and the power of all the methods increased in both the heritability and MAF values. In terms of the average power, MDR was 0.473, FMDR was 0.487, GMDR was 0.519, and FGMDR was 0.533. In the Wilcoxon signed-rank tests, the power of FGMDR was significantly higher than that of MDR (*p*-value: 1.31e-07), FMDR (1.36e-07) and GMDR (p-value: 1.94e-03).

### Real data experiments

#### Crohn’s disease (CD)

The CD data in Wellcome Trust Case Control Consortium [28] dataset, consists of 1949 cases and 3004 controls. For each individual, genetic information for about 500,000 SNPs, age information (in decades), and sex were provided. However, all values of the age information in the case samples, were the same value. Therefore, we used only sex as a covariate. For adapting our FGMDR method to analyze CD data, residuals were calculated, using the logistic regression model, with sex as a covariate and odds ratio of sex is 1.47 (95% confidence interval: 1.31–1.65, *p*-value of likelihood ratio test: 6.93E-11). Among SNPs, we selected 30 SNPs reported to associate with the CD phenotype [28, 30, 31] for illustration, and the basic characteristics of those SNPs, are summarized in Table 1. *P*-values and their rank of Table 1 were calculated by likelihood ratio test, under a codominant model with two degrees of freedom.

**Table 1:** Basic characteristics of each SNPs for CD

Index	rs number	MAF	Chromosome (gene)	p-value (rank)	Index	rs number	MAF	Chromosome (gene)	*p*-value (rank)
1	rs11805303	0.347	1 (IL23R)	1.41E-12 (2)	16	rs1456893	0.304	7	2.54E-05 (18)
2	rs12035082	0.410	1	4.34E-07 (9)	17	rs4263839	0.313	9 (NFSF15)	1.53E-05 (17)
3	rs10801047	0.079	1	7.31E-06 (15)	18	rs17582416	0.363	10 (OC105376492)	1.28E-03 (23)
4	rs11584383	0.297	1 (MROH3P)	3.71E-05 (20)	19	rs10995271	0.413	10	1.28E-05 (16)
5	rs3828309	0.453	2 (ATG16L1)	7.57E-14 (1)	20	rs10883365	0.498	10 (INC01475)	2.56E-06 (12)
6	rs9858542	0.299	3 (BSN)	2.50E-07 (8)	21	rs7927894	0.408	11	1.50E-02 (28)
7	rs17234657	0.146	5	4.90E-12 (3)	22	rs11175593	0.017	12 (OC105369735)	5.71E-02 (30)
8	rs9292777	0.367	5	2.02E-11 (4)	23	rs3764147	0.222	13 (LACC1)	3.78E-06 (13)
9	rs10077785	0.220	5 (C5orf56)	5.00E-05 (22)	24	rs17221417	0.310	16 (NOD2)	5.44E-10 (5)
10	rs13361189	0.084	5	5.96E-08 (6)	25	rs2872507	0.491	17	1.36E-03 (24)
11	rs4958847	0.130	5 (IRGM)	9.19E-07 (10)	26	rs744166	0.422	17 (STAT3)	4.99E-05 (21)
12	rs11747270	0.099	5 (IRGM)	2.54E-05 (19)	27	rs2542151	0.181	18	2.04E-07 (7)
13	rs6887695	0.329	5	6.86E-03 (27)	28	rs1736135	0.412	21 (LOC101927745)	2.98E-02 (29)
14	rs6908425	0.214	6 (CDKAL1)	1.01E-06 (11)	29	rs2836754	0.374	21 (LOC400867)	6.03E-06 (14)
15	rs7746082	0.293	6	4.13E-03 (26)	30	rs762421	0.408	21 (LOC105377139)	3.46E-03 (25)

We next performed FGMDRs with/without covariate adjustment, with 10-fold cross validation, from two to five-locus SNP combinations, as summarized in Table 2. FGMDR without covariate adjustment is performed to investigate the effect of covariate adjustment. SNP5 was consistently included the best SNP combinations from two to five-locus SNP combinations in the results of FGMDR with covariate adjustment, while SNP5 was included only for three and four-locus SNP combinations in the results of FGMDR without covariate adjustment. SNP combinations in three and four-locus models are the same in results of FGMDR with/without covariate adjustment but they are different in two and five-locus models. CVC values in the FGMDR with covariate adjustment are higher than or similar to that of FGMDR without covariate adjustment. BA values are similar in FGMDR with covariate adjustment and FGMDR without covariate adjustment regardless of training or testing data.

**Table 2:** Results of CD data analysis

order	FGMDR (with covariate adjustment)			FGMDR (without covariate adjustment)				
	SNP combination	CVC	*BA*_*FUZZY*_		SNP combination	CVC	*BA*_*FUZZY*_	
			training	testing			training	testing
2	5, 7	6	0.545	0.544	1, 8	5	0.545	0.542
3	1, 5, 7	6	0.561	0.554	1, 5, 7	4	0.561	0.554
4	1, 2, 5, 8	5	0.581	0.561	1, 2, 5, 8	3	0.581	0.561
5	5, 18, 19, 24, 28	3	0.612	0.560	1, 2, 3, 4, 19	2	0.612	0.560

*CVC* cross-validation consistency

**Fig. 4:**
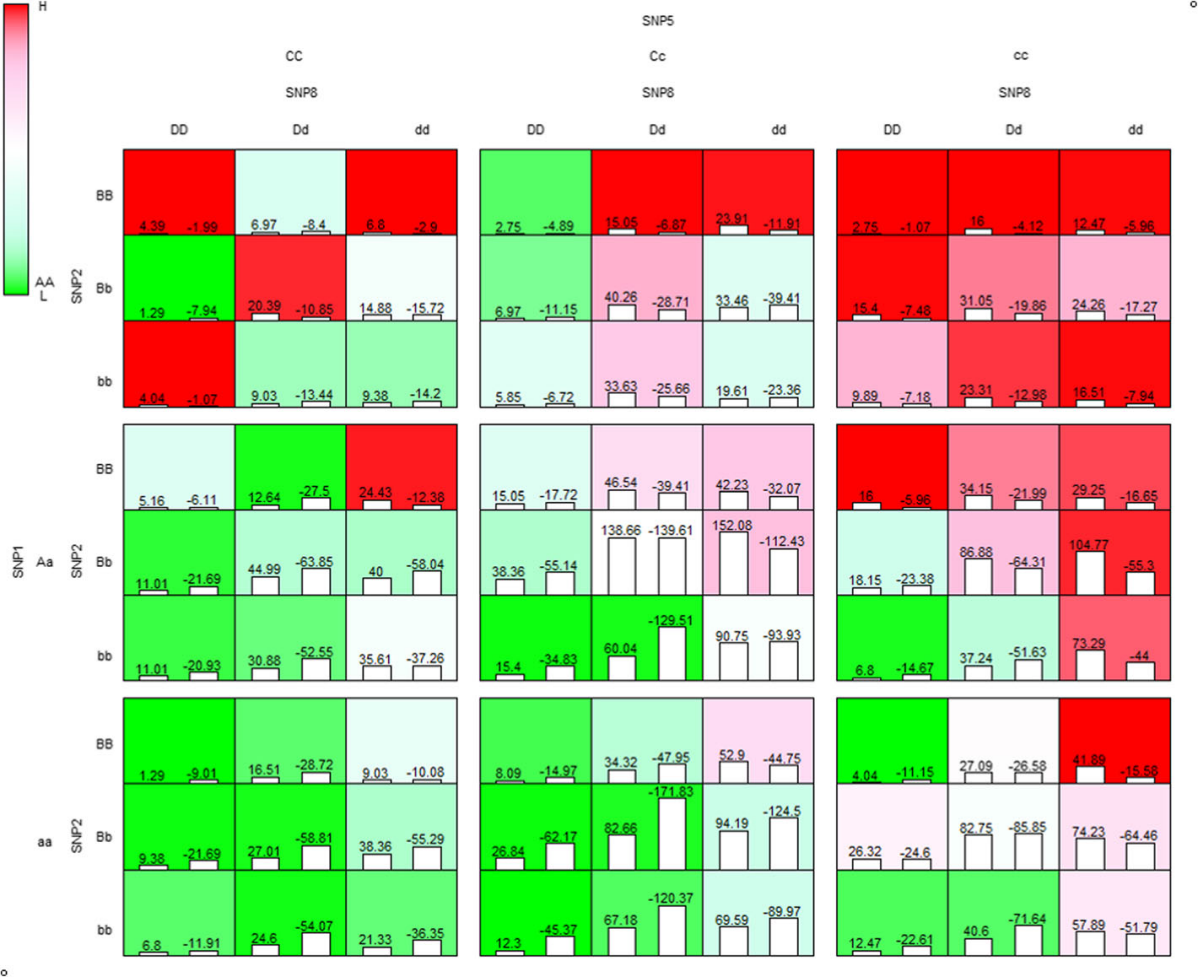
Interpretation of the 4-locus SNP interaction in the result of CD

Among these results, we selected a four-locus (order: 4) SNP combination as the best SNP combination, based on the best *BA*_*FUZZY*_ in testing data, and its relatively high cross-validation consistency (CVC) value. Interaction of this SNP combination is represented in Fig. 4.

In Fig. 4, upper case letters denote major alleles, while and lower case letters denote minor alleles. ‘A’ or ‘a’ represent the genotypes of the first SNP in the SNP combination; ‘B’ and ‘b’ represent the genotypes of the second SNP, and so on. The left bar-labeled value represents the sum of the positive residuals, while the right bar-labeled value represents sum of the negative residuals. The green background colored cells mean the membership value that cells are close to 0, and the red background colored cells mean the membership value that cell are close to 1. The dark background color means the value is far from 0.5 (i.e., closer to 1 or 0), while the white background color denotes a 0.5 membership value.

Figure 4 shows some interesting patterns for interpretation of the interaction. First, with respect to the diagonal line, most of the cells in the right top quadrant had red background, while most of the cells in the lower-left quadrant cells were green background. Based on these observations, it seems that an additive risk pattern increased from left to right, and from top to bottom. However, genotype patterns represent combinations of two SNPs with vertical and horizontal genotypes. For example, in vertical genotypes, there are genotype combinations of SNP1 and SNP2. However, note that the order of the SNP combination is important for interpretation. For example, (SNP1, SNP2) seemed to be additive in effect, while (SNP2, SNP1) didn’t suggest an additive effect. A possible interpretation of interaction between SNP1 and SNP2 is that the risk of CD is dominated by SNP1 minor allele at first and SNP2 then affects CD risk for each genotype sample of SNP1. Second, interaction patterns of SNP2 and SNP8 are not consistent for each genotype combination of SNP1 and SNP 5. The interaction patterns of SNP2 and SNP8 are represented by separated blocks, consisting of 3 × 3 cells. For example, the color pattern of the top left block (SNP1, SNP5) = (AA, CC) is different from all other blocks.

Additionally, the interaction between IR23R (SNP1) and ATG16L1 (SNP5) for CD, was reported and in a case-control study within a cohort study [32], and reviewed for explanation of CD mechanism [33]. However, we cannot find direct evidence of interactions of these particular SNPs in the four-SNP combination.

### Homeostatic model assessment of insulin resistance (HOMA-IR)

We next analyzed HOMA-IR data from the Korea Association REsource project (KARE) to illustrate FGMDR in the context of quantitative traits. A total of 8577 samples are available, after removing subjects with at least one missing phenotype value. The genomic DNAs were genotyped using Affymetrix Genome-Wide Human SNP Array 5.0. For GGI analysis using our FGMDR, we used only 10 candidate SNPs identified in earlier studies [34–36] from the single SNP GWAS analysis. The basic characteristics of these SNPs are summarized in Table 3. *P*-values and their rank in Table 3, were calculated by likelihood ratio test, under a codominant model with two degrees of freedom. Since the distribution of HOMA-IR is skewed, many researchers perform a log-transformation before applying the regression analysis [34], and we did likewise. Sex, age, area, and BMI were used as environmental covariates. Then, the regression model for FGMDR is given by

2 $$ \log \left(\mathrm{HOMA}-\mathrm{IR}\right)={\beta}_0+{\beta}_1{Sex}_i+{\beta}_2{Age}_i+{\beta}_3{Area}_i+{\beta}_4{BMI}_i+{\varepsilon}_i. $$

Using the residuals calculated from (2), FGMDR was then performed.

**Table 3:** Basic characteristics of each SNPs for HOMA-IR

Index	rs number	MAF	Chromosome (gene)	p-value (rank)	Index	rs number	MAF	Chromosome (gene)	p-value (rank)
1	rs4915657	0.405	1(ROR1)	2.12E-3(6)	6	rs702634	0.109	5(ARL15)	3.40E-1(10)
2	rs576563	0.338	1(JAK1)	9.85E-4(3)	7	rs7754840	0.476	6(CDKAL1)	1.80E-1(9)
3	rs693	0.056	2(APOB)	5.45E-3(7)	8	rs9353581	0.455	6	1.90E-3(5)
4	rs780094	0.463	2(GCKR)	1.60E-2(8)	9	rs2920792	0.417	10	5.44E-4(2)
5	rs11125090	0.273	2	1.12E-5(1)	10	rs7500315	0.416	16	1.20E-3(4)

We performed FGMDRs with/without covariate adjustment, with 10-fold cross validation from two to five-locus SNP combinations and summarized the results of HOMA-IR in Table 4. All best SNP combinations included SNP 5 except 5 locus SNP combination with covariate adjustment, consistent with its *p*-value in Table 3 (the lowest p-value and rank: 1). In the results of FGMDR without covariate adjustment, SNPs in lower order SNP combination models are included in higher order SNP combination models. In addition, BA values of FGMDR with covariate adjustment are higher than those of FGMDR without covariate adjustment in both training and testing data. These differences may be caused by covariate adjustment. Similar to the results of CD data analysis, SNP combinations identified by FGMDR with covariate adjustment are different from those by FGMDR without covariate adjustment. While a further biological investigation is required, we expect that the covariate adjustment makes not only a performance improvement but also a more accurate identification of true causal SNP interactions.

**Table 4:** Results of HOMA-IR data analysis

order	FGMDR (with covariate adjustment)			FGMDR (without covariate adjustment)				
	SNP combination	CVC	*BA*_*FUZZY*_		SNP combination	CVC	*BA*_*FUZZY*_	
			training	testing			training	testing
2	5, 9	10	0.513	0.510	5, 9	10	0.512	0.509
3	5, 8, 9	6	0.522	0.514	5, 8, 9	7	0.521	0.512
4	1, 2, 5, 10	5	0.539	0.517	5, 8, 9, 10	6	0.537	0.513
5	1, 2, 4, 7, 8	4	0.572	0.516	5, 7, 8, 9, 10	6	0.571	0.514

*CVC* cross-validation consistency

Among the results of FGMDR, we selected the four-locus SNP combination as the best SNP combination based on *BA*_*FUZZY*_ in testing data and CVC. Three SNPs in the selected SNP combinations except SNP10 are located in ROR1, JAK1, and nearby SOCS5 (about 19.8 k BP). For these three genes, several biological evidences of interactions are pre-reported: 1) ‘Jak1 has previously been implicated in adipocyte insulin resistance.’ [37], 2) ‘Most of the known SOCS proteins are involved in the modulation of the development of insulin resistance.’ [38], 3) ‘When JAK1 and SOCS5 are co-expressed in cells, JAK1 is continually being phosphorylated and de-phosphorylated during the course of the transfection, and SOCS5 presumably interacts with active (phosphorylated) JAK1 to inhibit further enzymatic activity’ [39], 4) ‘ROR1 was shown to interact with and be inhibited by resistin.’ [40], 5) ‘Resistin is also correlated with insulin resistance.’ [41].

CVCs decreased by increase of order, in both Tables 2 and 4. This is a general phenomenon in multi-locus association tests. For example, among 30 SNPs, there are 435 possible two-locus SNP combinations and 2610 possible three-locus SNP combinations. An interesting point is relatively low *BA*_*FUZZY*_ in testing. These *BA*_*FUZZY*_ values are not directly comparable to ordinary *BA* because the Fuzzy set theory has been implemented. *BA*_*FUZZY*_ is more concentrated near 0.5, compared to ordinary *BA*. Nevertheless, *BA*_*FUZZY*_ values in testing HOMA-IR data were lower than those of CD. This seems to be caused by heritability differences. The heritability of CD is 53% [42] but the heritability of HOMA-IR is 8% in black and Spanish populations [43], and 22% in Asian Indian families [44].

## Discussion and Conclusion

In this study, we proposed a FGMDR, a fuzzy extension of GMDR to detect GGIs. FGMDR can handle both binary and quantitative traits, and allows adjustment for covariates. Thus, FGMDR is a method to overcome shortcomings due to the traditional classification commonly used in MDR-based frameworks by allowing partial membership degrees of high and low risk groups for each cell, and provides more flexible interpretations for results. Our proposed FGMDR is based in the generalized linear models (GLMs), it can handle any distributions of phenotypes from the exponential family including normal, binomial, Poisson and gamma distributions. Further, our FGMDR does not require any balancing of H/L risk groups. Three simulation scenarios for power comparison were made under one continuous phenotype, and two binary phenotypes with adjustment for covariates. Simulation studies showed that FGMDR outperformed MDR and GMDR in most scenarios. Through two real applications to CD and HOMA-IR data, we identified the best SNP combinations associated with two diseases. In our applications, we found several biological evidences of two-order interactions included high-order interactions identified by FGMDR (four-way interaction for CD and four-way interaction for HOMA-IR).

The existing MDR extensions using classical sets as groups for classification, can be extended to any fuzzy set-based MDR methods. These fuzzy set-based MDR methods may contribute to identify important interactions in the biological systems, through reflecting the vagueness of classification due to objects that can seldom be classified uniquely.
